# Measuring spatial accessibility to healthcare services with constraint of administrative boundary: a case study of Yanqing District, Beijing, China

**DOI:** 10.1186/s12939-018-0720-5

**Published:** 2018-01-15

**Authors:** Zhuolin Tao, Yang Cheng, Qingjing Zheng, Guicai Li

**Affiliations:** 1 College of Urban and Environmental Sciences, Peking University, Beijing, 100871 China; 2 School of Urban Planning and Design, Peking University, Shenzhen, Guangdong 518055 China; 3 School of Geography, Faculty of Geographical Science, Beijing Normal University, 19 Xinjiekouwai Street, Haidian, Beijing, 100875 China; 4 China Academy of Urban Planning and Design Shenzhen, Shenzhen, Guangdong 518034 China

**Keywords:** Spatial accessibility, Administrative boundary, 2SFCA, Catchment area, Healthcare service, Beijing

## Abstract

**Background:**

The two-step floating catchment area (2SFCA) method, which is one of the most widely used methods for measuring healthcare spatial accessibility, defines the catchment area of each facility as the area within a certain distance from the facility. However, in some cases, the service utilization behavior is constrained by administrative boundaries, therefore the definition of catchment area within a certain distance may be inappropriate.

**Methods:**

In this study, we aim to propose a modification of the 2SFCA method for measuring spatial accessibility to healthcare services in a system constrained by administrative boundaries. The proposed method defines the catchment areas of healthcare facilities within certain administrative units. The method is applied in a case study of the healthcare services in Yanqing District of Beijing, China. Three types of healthcare facilities, including general hospitals, community healthcare centers and stations, are included.

**Results:**

Based on the sensitivity analysis of the distance-decay parameter *β*, result of the *β* = 1 scenario is relatively appropriate and is utilized for further analysis. The difference between spatial accessibility with or without constraint of administrative boundary is relatively significant. The results of the proposed model show that the village-level spatial accessibility to healthcare services shows a significant disparity, and the uneven distribution of general hospitals is the main cause.

**Conclusions:**

The constraint of administrative boundary has a significant impact on healthcare accessibility, which verifies the validity of the modification proposed by this study in empirical studies. The empirical results also lead to policy recommendations to improve healthcare equity in the study area. At the town-level, the improvement of equity in healthcare accessibility could be achieved in two ways. First, the sizes of community healthcare centers in towns with small accessibility scores should be expanded. Second, new general hospitals can be built in the eastern part of Yanqing District. Within each town, to improve the equity in healthcare accessibility, community healthcare stations should be expanded or newly built in the periphery villages.

## Background

Spatial accessibility has been widely applied as a scientific method for assessing the distribution of public services such as healthcare services [1–3]. It can be used to measure the disparities in the opportunities to access healthcare services among various population groups or regions [2, 4]. Among the methods for measuring spatial accessibility to healthcare services, the two-step floating catchment area (2SFCA) method is one of the most widely used methods [2, 4]. However, in the definitions on catchment area in traditional 2SFCA method and the existing variants, interactions between demanders and facilities can cross administrative boundaries, which is not appropriate in some cases. This study aims to propose a modified form of 2SFCA method for measuring spatial accessibility to healthcare services in a system constrained by administrative boundary.

Spatial accessibility emphasizes either or both of the following two factors: one is the spatial impedance between demand and supply, and the other is the availability of services (i.e. the amount of supply that is available to a population group) [1]. Distance to the closest facility and ratio of supply to demand are two simple traditional measures [5]. The former emphasizes the spatial impedance a demander has to overcome to obtain services, but it is not taken into account the capacities of facilities and the possibility that a demander visits multiple facilities [6, 7]. In contrast, the measurement of supply-demand ratio emphasizes the availability of services by calculating ratio of supply to demand in a certain region (usually defined as administrative units), but it fails to take into account the spatial impedance [1].

Some more comprehensive methods have been developed for measuring spatial accessibility. Hansen [8] proposed a gravity-based potential model to measure accessibility to jobs in 1959, which considers both the spatial impedance between demand and supply and the capacity of facilities. Joseph et al. [9] improved the potential model by further incorporating the factors on the demand side. 2SFCA method was first proposed by Radke et al. [10] and named by Luo et al. [11]. Essentially, 2SFCA method is a kind of gravity-based measure, though it takes a dichotomous distance-decay function [11]. Various improvements have been developed for the basic form of 2SFCA, such as improvements in various (discrete or continuous) distance-decay functions [12–14], variable catchment areas [15–17], the competition effects among demanders or facilities [18–20], and improvements that incorporate multiple transportation modes [21, 22]. All of these improvements greatly strengthen the rationality of 2SFCA method and its applicability for different scenarios.

In the 2SFCA method, the catchment area of a facility is defined as the area within a certain physical distance/travel time from the facility [23]. Most variants of 2SFCA use this definition of catchment area following the original form of 2SFCA. Jamtsho et al. [23] proposed a new nearest-neighbor method for delineating catchment areas, which assumes each demand node only selects a finite number of nearest facilities. In the above two kinds of definitions on catchment area, interactions between demanders and facilities can cross administrative boundaries.

However, in some cases, the service utilization behavior may be constrained within administrative boundaries. For example, the primary healthcare services in China are usually provided within a certain level of administrative boundaries, which the medical insurance is tied to. The nine-year compulsory education in China is another example. The compulsory education services are provided at the school district’s level which are usually in accord with administrative boundaries in rural China. Such cases can also be found in other countries (including western countries). For example, the catchment areas of hospitals in Finland are divided based on administrative districts [24]. In such cases, the catchment areas of facilities should be linked to administrative boundaries. If the constraint of administrative boundary is neglected in such cases, the result of accessibility analysis would be biased.

To date, the constraint of administrative boundary on healthcare accessibility has not been considered in existing improvements of 2SFCA method. This study aims to contribute to the literature by filling this gap. A modified form of 2SFCA is developed, within which services of a facility can only be accessed and utilized by demanders within a certain administrative boundary. The proposed model will be applied for measuring the spatial accessibility to the healthcare services in Yanqing District of Beijing, China. A comparison will be conducted between the results of the proposed model and the common 2SFCA model, to reveal the impact of the constraint of administrative boundary on accessibility results. The empirical findings could provide knowledge-based suggestions for the decision-making of healthcare service allocation in Yanqing District. The proposed method could be applied for measuring spatial accessibility of other types of public services and in other regions.

## Methods

### The generalized 2SFCA framework

The 2SFCA method measures the spatial accessibility by two steps. In the first step, it searches all the demand nodes within the catchment areas of each facility, then calculates supply-to-demand ratios for each facility, i.e. the average supply per potential service user. In the second step, it adds up the supply-to-demand ratios of all facilities located in the catchment area of each demand node. The sum of supply-to-demand ratios for each demand node is its spatial accessibility score. The original form of 2SFCA adopts a dichotomous distance decay function, supposing the demanders within catchment areas share equal accessibility while the demanders outside catchment areas are completely inaccessible to services. Several extensions of 2SFCA have been developed with respect to the distance-decay function. Different forms of distance-decay functions are incorporated into the 2SFCA framework, which can be uniformly expressed by a generalized 2SFCA framework (G2SFCA) [4]. It is mathematically succinct but integrates the potential model and 2SFCA with various distance-decay functions. Therefore, our analysis is built on the basis of the G2SFCA framework. It can be expressed as:

1 $$ {A}_i=\sum \limits_j\frac{S_jf\left({d}_{ij}\right)}{\sum \limits_k{P}_kf\left({d}_{kj}\right)} $$

where *A*_*i*_ is the accessibility at demand node *i*, *S*_*j*_ is the capacity of supply at location *j*, *P*_*k*_ is the demand amount, *d*_*ij*_(*d*_*kj*_) is the distance or travel time between *i*(*k*) and *j*, *f* is a general distance-decay function. Specifically, *A*_*i*_ refers to the amount of accessible supplies per demander at the demand node *i*.

The distance-decay function *f* can take various forms, such as the dichotomous form of the original 2SFCA [11], the Gaussian form [13] and the kernel density form [14]. In this study, the gravity-style power function is adopted as the distance-decay function, following Wang et al’s and Tao et al’s studies [25, 26]. The power function is widely employed by gravity model in spatial interaction modelling and has superiority in mathematical simplicity of modelling compared with other alternatives. The distance-decay function *f* can be written as:

2 $$ f\left({d}_{ij}\right)=\left\{\begin{array}{c}{d}_{ij}^{-\beta },\kern0.5em {d}_{ij}\le {d}_0\\ {}\kern0.5em 0,\kern1em {d}_{ij}>{d}_0\end{array}\right. $$

where *d*_*ij*_ is the distance or travel time between *i* and *j*, *β* is the distance-decay parameter, *d*_0_ is the size of catchment area of facilities.

### Modeling constraint of administrative boundary

A modification with respect to the delineation of catchment areas is made based on the generalized 2SFCA framework, to model the constraint of administrative boundary on spatial accessibility. The modified model can be written as:

3 $$ {A}_i=\sum \limits_{j\in {F}_i}\frac{S_jf\left({d}_{ij}\right)}{\sum \limits_{k\in {D}_j}{P}_kf\left({d}_{kj}\right)} $$

where *F*_*i*_ denotes the set of facilities within the catchment area of demand node *i*, *D*_*j*_ denotes the set of demand nodes within the catchment area of facility *j*, and other variables confirm to formula (1). Particularly, the catchment area is delineated by a certain level of administrative boundaries. In another word, *F*_*j*_ denotes the set of facilities within the same administrative unit with the demand node *i*, and *D*_*j*_ denotes the set of demand nodes within the same administrative unit with the facility *j*. From this, the distance-decay function can be modified as:

4 $$ f\left({d}_{ij}\right)=\left\{\begin{array}{c}{d}_{ij}^{-\beta },\kern0.75em j\in {F}_i\  or\ i\in {D}_j\\ {}0,\kern2em otherwise\end{array}\right. $$

where all variables are the same with formula (2) and (3). The distance-decay parameter *β* can take various values by conducting a sensitivity analysis, since any single value of *β* is more or less arbitrary.

**Fig. 1:**
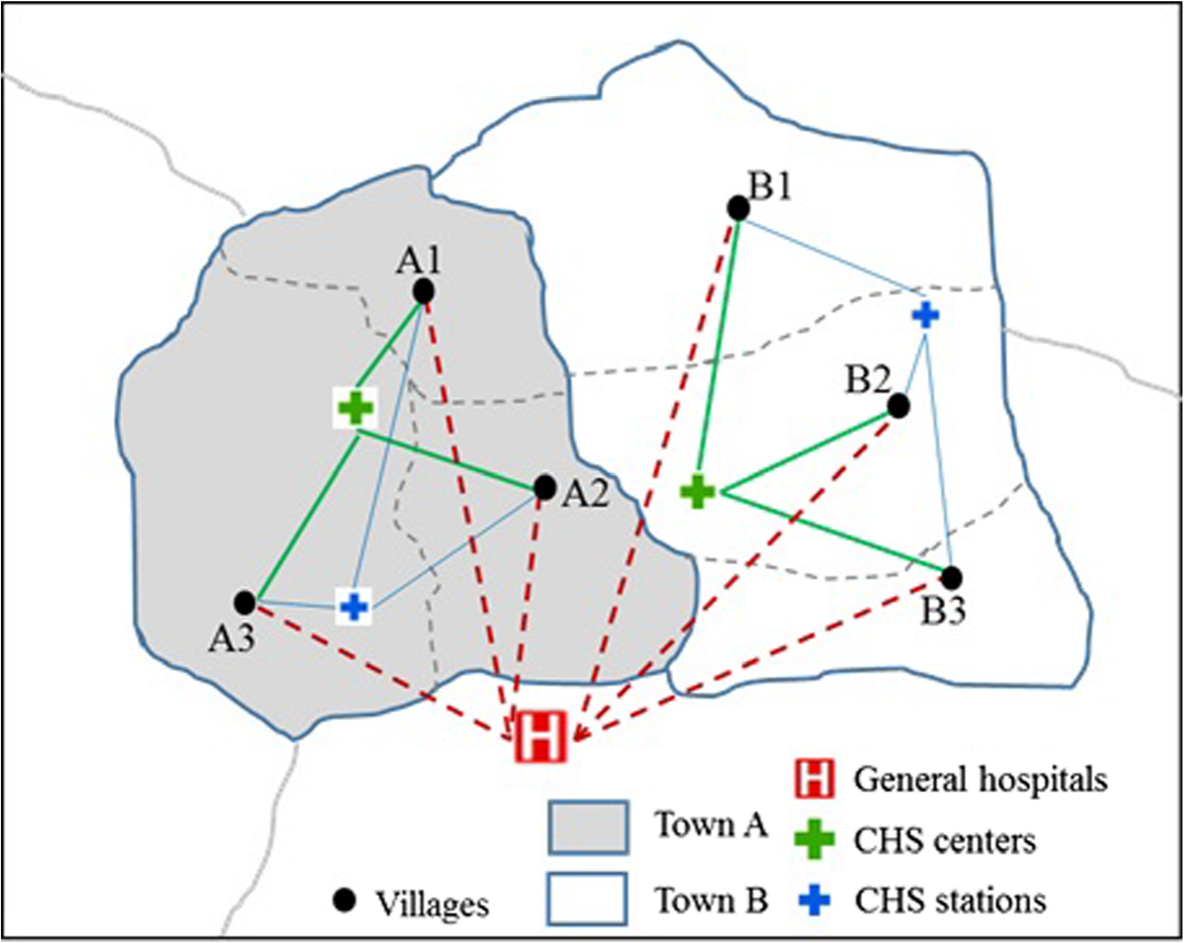
Illustration graph of the catchment areas delineated by administrative boundaries

The method can be demonstrated by Fig. 1. There are three levels of healthcare facilities in this case, the general hospitals (GHs), the community healthcare centers (CHCs), and the community healthcare stations (CHSs), which is the typical healthcare system in the suburban and rural areas in China. The GHs provide healthcare services for the whole district. The CHCs are configured at the township level. Each CHC covers the demanders in the whole town. The CHSs, corresponding to the lowest level, are configured at the village level. Theoretically, the CHSs should be located in each village. In reality, however, not every village has a CHS due to geographical and economic reasons. Therefore, CHSs have to provide healthcare services across village boundaries but within the town-level boundaries.

In this case, there are two-level administrative boundaries delineating the catchment areas of healthcare facilities. The town-level boundaries delineate the catchment areas of CHCs and CHSs, and the district-level boundaries delineate the catchment areas of the general hospitals.

In the case presented in Fig. 1, each of the two towns (A and B) has a CHC, while only village A3 and village B2 have CHSs. Both CHCs and CHSs can be accessed by all villages within the town-level administrative boundaries. The catchment of the GH covers the whole district. Therefore, though the GH is located outside of the town A and B, the villages in both towns can access to the services provided by the GH. In the demand node side, taking village A2 as an example, it can access one CHS and one CHC in town A as well as the GH outside of the town A. However, A2 cannot access to the CHC in town B, though it is quite close to A2.

In this study, the calculations of both the traditional and modified 2SFCA method are conducted by programming in MATLAB (version 2015a).

## Case study

### Study area

**Fig. 2:**
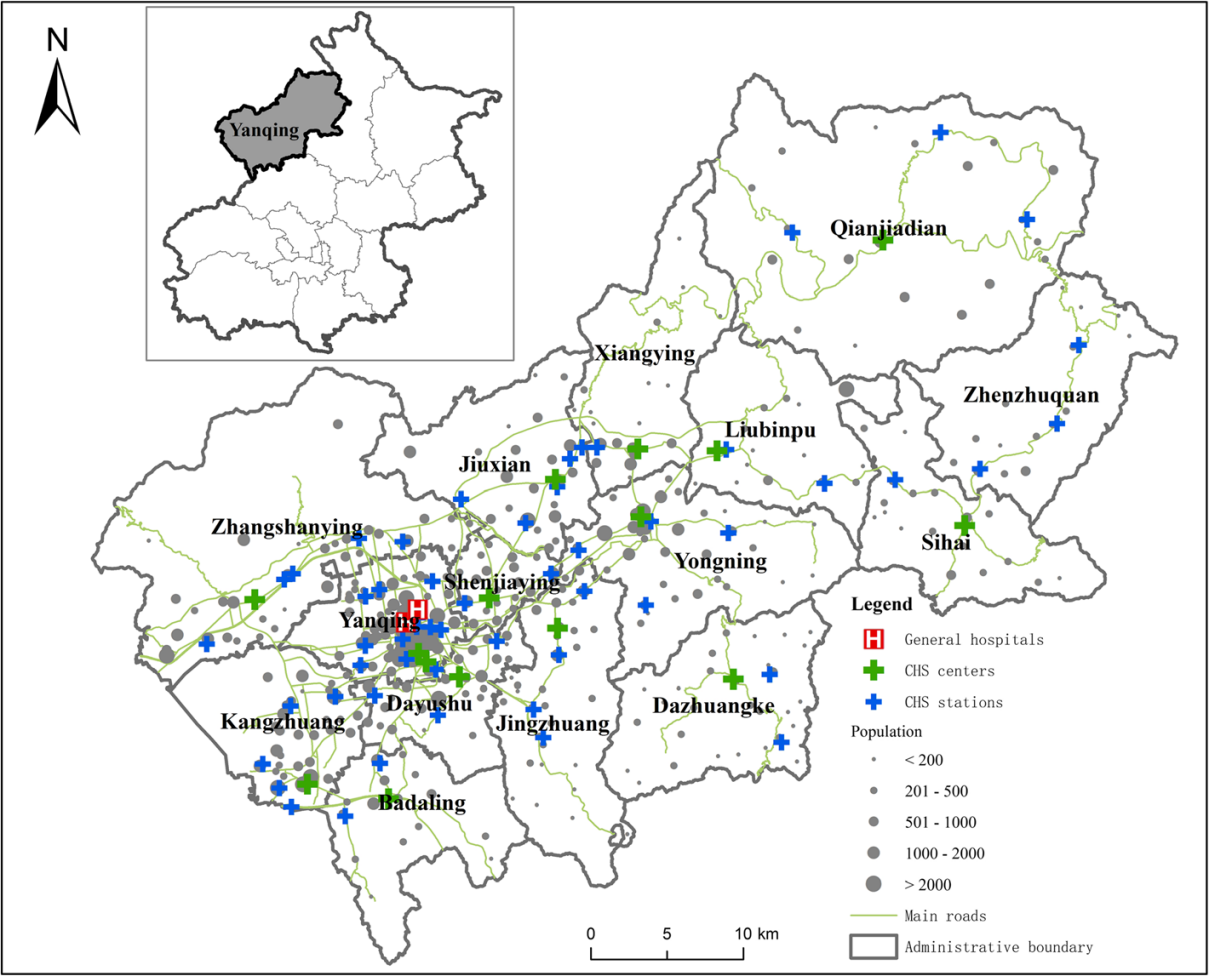
Distribution of population and healthcare facilities in Yanqing District

Yanqing District is located in the northwest of Beijing. Its area size is 1994 km^2^ and the permanent population was 316,000 in 2014. The whole district is constituted by 15 subdistrict-level administrative units (i.e. towns). The district center, where the local district government is located, is in Yanqing Town (Fig. 2).

The list of the healthcare facilities as well as their locations and numbers of physicians are obtained from the website of the Health and Family Planning Commission of Beijing Yanqing District [27]. The list was updated in July, 2016. The village-level population data are from the sixth census data in 2010 provided by the local government, which is the latest available population data at the village level.

There are three levels of healthcare facilities in Yanqing. Each level of healthcare services is corresponding to a certain level of administrative boundary. There are two general hospitals in Yanqing, namely, the People’s hospital of Yanqing and the Yanqing traditional Chinese medicine hospital, providing healthcare services for the whole district. There are 15 CHCs in Yanqing. Among the 396 villages, 54 villages have CHSs. The CHCs and CHSs provide services within the town boundary in which they are located. Therefore, the situation in Yanqing District is a typical example in Fig. 1. The service capabilities of these healthcare facilities are represented by the number of physicians.

### Road network processing

The road network is digitized by using ArcGIS (version 10.3) based on existing main roads in the comprehensive transportation planning map of Yanqing District. Using the constructed road network, we utilize the OD Matrix tool in Network Analysis toolbox of ArcGIS to calculate the nearest travel distance from each demand node to each facility via the road network as travel cost to access to healthcare services. Note that this road network connects most major settlements, but it is incapable of connecting a considerable part of villages, which are also demand nodes adopted in this study.

In addition, the OD Matrix tool ignores the distance from endpoints to road network. In reality, however, all villages (demand nodes) and healthcare facilities should be connected to road network by either high-level or low-level roads. Therefore, travel distance departing from/arriving to those endpoints (demand nodes/facilities) not connected to any road would be omitted. As a result, the estimated travel distance would be under-estimated. The further the endpoint is away from the nearest road, the more significant the under-estimation is. The reason is that the obtained road network data does not contain all roads in reality, especially those low-level roads, thus failed to connect all demand nodes and facilities.

To address the under-estimation problem, we use the ‘connecting to lines’ tool of TransCAD software (version 4.5) to generate new lines connecting the separated endpoints to road network. The new lines generated by ‘connecting to lines’ tool are then integrated into the road network. By doing so, all endpoints are connected to road network, and the distance from endpoint to road network is taken into account.

### Scenario setting

Due to lack of relative information to set a best value of the distance-decay parameter *β*, a sensitivity analysis is needed to compare accessibility results for different *β* values. Two scenarios where *β* = 1 and 2 are employed to investigate the impact of parameter values on results of this study.

In addition, to investigate the impact of constraint of administrative boundaries on accessibility patterns, a comparison between the accessibility results of the proposed model (with constraint) and the results of the common model (without constraint) are carried out. To conduct an analysis of the common 2SFCA model, in addition to the distance-decay parameter *β*, the size of catchment area is also required to assign a value. For the convenience of comparison, the value of *β* is set as 1, and the result is compared with the scenario of with-constraint when *β* = 1. The size of catchment area should be comparable with the scenario of with-constraint. As for the GHs, the catchment area is set as the whole scope of the study area, which is the same as the scenario of with-constraint. As a result, the difference between the accessibility of two scenarios is actually the difference between accessibility to CHCs and CHSs. As for the CHCs and CHSs, the size of catchment area is set according to the average area of townships (132.7 km^2^). If assuming the shape of a township is circle, the radius corresponding to the average area is 6.5 km. Therefore, the size of catchment area of CHCs and CHSs is set as 6.5 km, which is comparable with the with-constraint scenario.

## Results

### Results of overall accessibility

**Fig. 3:**
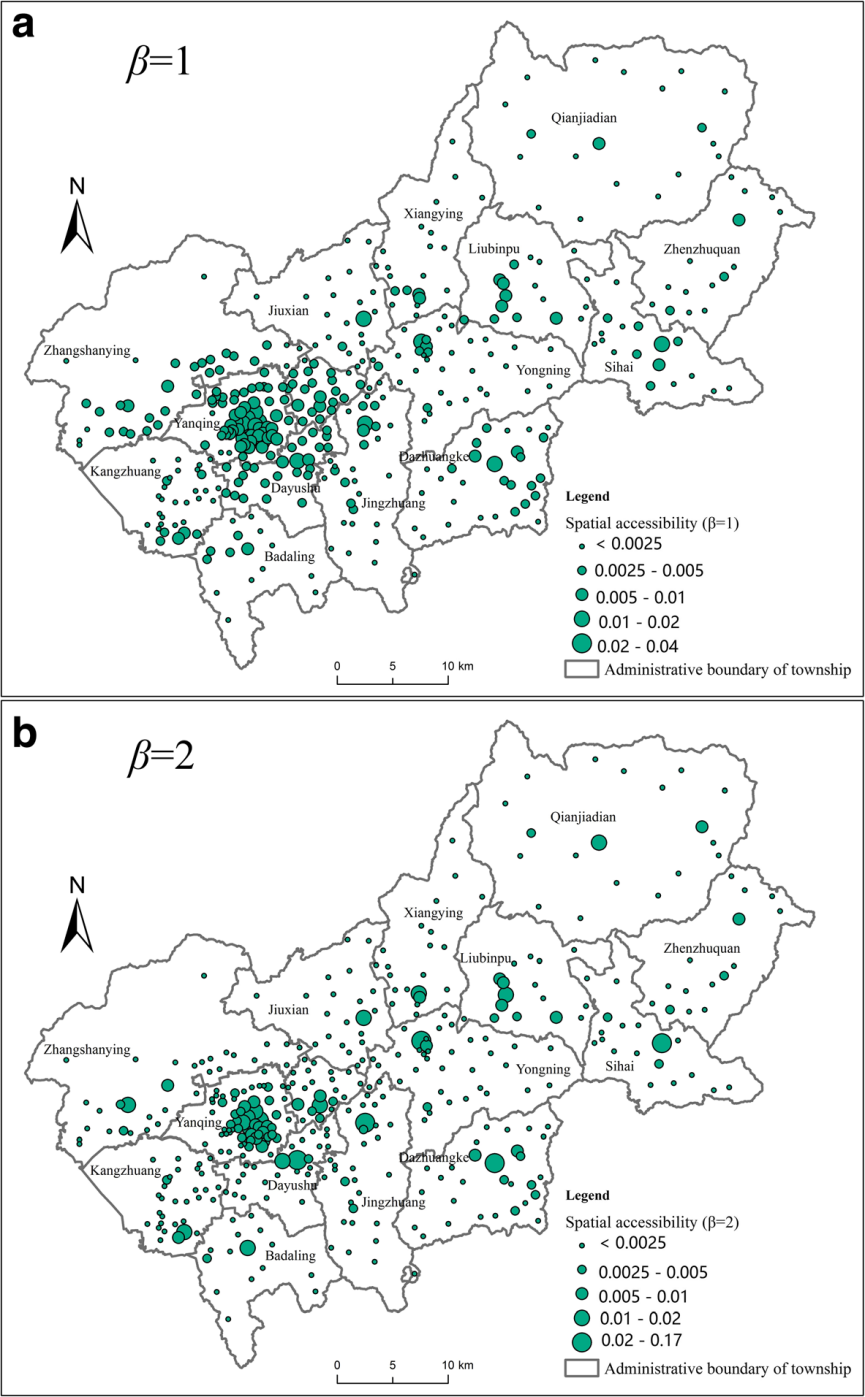
Spatial accessibility to healthcare facilities in Yanqing District when (**a**) β=1 or (**b**) β=2

Based on the village-level population data and the existing three-level healthcare facilities, the proposed method has been applied to measure the spatial accessibility to healthcare facilities in Yanqing District. Two scenarios, where the value of distance-decay parameter is 1 and 2 respectively, are both calculated (Fig. 3).

The results show that the distributions of spatial accessibility to healthcare facilities in the two scenarios are quite similar, both showing significant differences among spatial accessibility at the village level. In both scenarios, the spatial accessibility in the surrounding area of district center, namely Yanqing Town and Shenjiaying Town, is prominently higher than other areas. Moreover, the village-level accessibility is relatively even in these two towns. One reason is that there are more CHCs and CHSs in this area, and these facilities spread out over the whole area, enabling all the demand nodes in the two towns access to considerable CHCs and CHSs. The other reason is the proximity to the two GHs enabling the demand nodes in the two towns have better accessibility to healthcare service to GHs than demand nodes in other towns.

In contrast, in the other 13 towns, the village-level spatial accessibility to healthcare service shows significant difference within each town. The majority of the villages have relatively poor accessibility to healthcare services except a few villages where the CHCs and CHSs are located. This is because of the uneven distribution of the CHCs and CHSs in each of the 13 towns, and healthcare resources are spatially aggregated within each town’s scope.

**Fig. 4:**
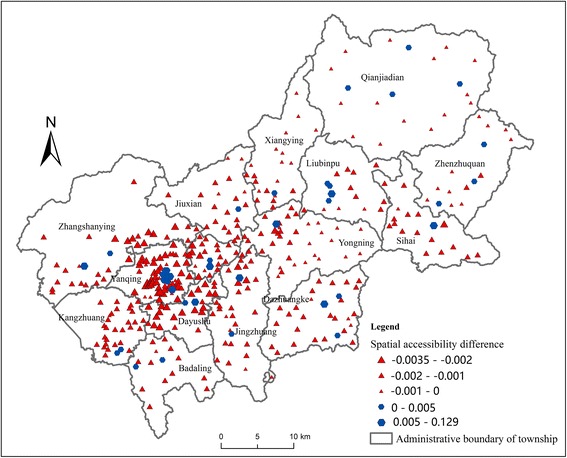
The difference in spatial accessibility between *β* = 1 scenario and *β* = 2 scenario

Besides the dominant similarity above, the results of two scenarios also present slight differences. In the *β* = 1 scenario, the range of accessibility scores varies from 0.0005 to 0.04 (Fig. 3a); while in the *β* = 2 scenario, the range increases dramatically, with a minimum of 0.001 and a maximum of 0.17 (Fig. 3b). To more explicitly compare the results of the two scenarios, the difference of each demand node’s accessibility is calculated (the accessibility *β* = 2 minus the accessibility *β* = 1). Thus, a positive difference indicates that the accessibility is better when *β* is larger. The accessibility differences are shown in Fig. 4. By comparing the distribution of accessibility differences and the distribution of healthcare facilities (Fig. 2), the results show that the positive accessibility differences are distributed in the areas close to the facilities. When the parameter *β* is larger, the demand nodes near facilities tend to have better accessibility scores. In another word, a larger *β* has a stronger distance-decay effect on the spatial accessibility, which confirms to the general knowledge and conclusions of most existing studies.

**Fig. 5:**
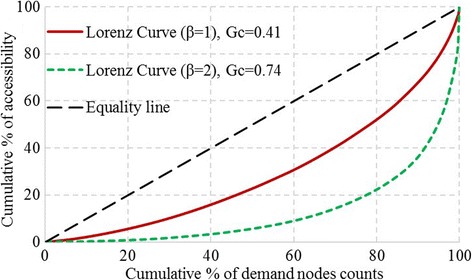
Lorenz curves of accessibility to healthcare services of two scenarios

The disparity of accessibility can be measured by calculating the Gini coefficient (Gc) corresponding Lorenz curve. The Lorenz curves of both scenarios are shown in Fig. 5. The equality line denotes a completely even distribution of accessibility to healthcare services, i.e. each demand node has an equal accessibility. A larger area between Lorenz curve and the equality line indicates a larger disparity in accessibility, as well as a larger Gini coefficient. It is shown in Fig. 5 that the Lorenz curve (*β* = 2) is further away from the equality line than the Lorenz curve (*β* = 1), and the corresponding Gini coefficients are 0.74 and 0.41 respectively. It indicates that the spatial disparities of accessibility in both scenarios reach a relatively high level. Furthermore, when the distance-decay parameter *β* is larger, the disparity of accessibility is more significant. It again confirms that a larger *β* means a stronger distance-decay effect on accessibility. The sensitivity analysis helps set the value of the parameter in accordance with the healthcare service utilization behavior.

Based on the sensitivity analysis, when *β* = 2, the disparity in accessibility is overly larger. Sixty percent of healthcare accessibility is assigned to less than 10% of demand nodes. In addition, demand nodes with the highest accessibility are highly concentrated surrounding the healthcare facilities. Therefore, results of the *β* = 2 scenario may overestimate the disparity of accessibility and the distance-decay effect in this case. In the following subsections, results of the *β* = 1 scenario will be utilized for further analysis.

### Distribution of CHC, CHS and GH accessibility

**Fig. 6:**
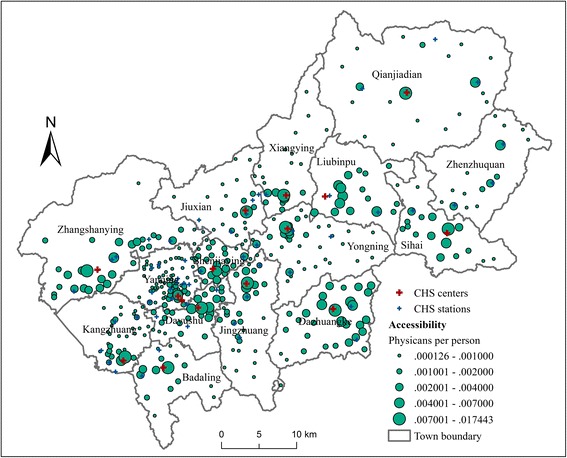
Accessibility to CHCs and CHSs in Yanqing District when *β* = 1

In this part, the accessibility to CHCs, CHSs and GHs are analyzed separately. Only the *β* = 1 scenario is analyzed based on the result of sensitivity analysis. Figure 6 shows the distribution of the accessibility to CHCs and CHSs in Yanqing District. The CHCs and CHSs in each town provide services for villages within the town boundaries. The distribution of accessibility within each town shows difference, but the degree of difference varies in each town. In towns with less CHCs and CHSs and more disperse distribution of villages, such as Qianjiadian, Zhenzhuquan, Xiangying, Jiuxian, Yongning and Jingzhuang, the spatial difference of accessibility is more significant. The accessibility is relatively high for residents in the villages located surrounding the CHCs and CHSs, while the villages far away from the CHCs and CHSs are with relatively poor accessibility. In contrast, in towns with more CHCs and CHSs and relatively concentrated contribution of villages, such as Shenjiaying, Liubinpu, Dazhuangke, Dayushu and Zhangshanying, the distribution of accessibility is relatively even. The distribution pattern of spatial accessibility shows that the total amount of health care resources in a town affect its spatial equity.

**Fig. 7:**
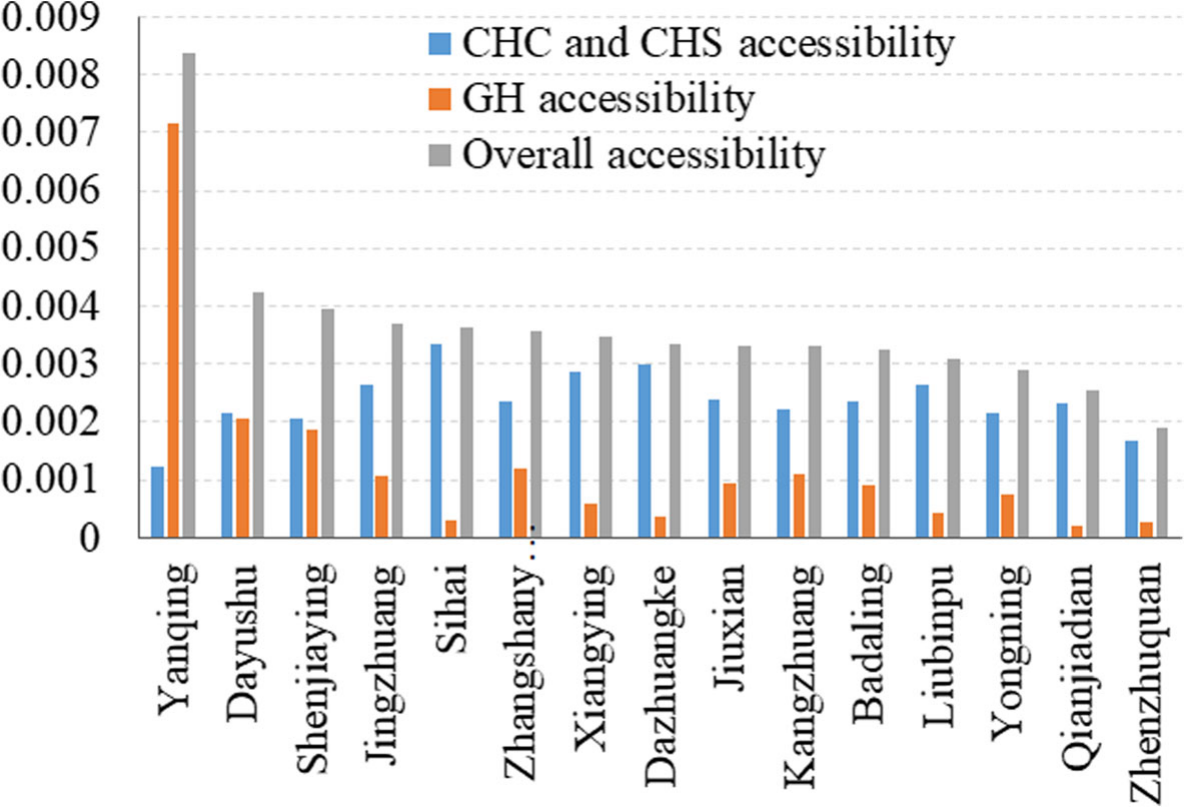
Average accessibility of towns to CHCs, CHSs and GHs when *β* = 1

The accessibility level of each town shows similar pattern in Fig. 6. A further comparison is conducted by calculating the population-weighted average accessibility of each town. As shown in Fig. 7, the weighted average CHC and CHS accessibility of each town ranges from 0.00122 (Yanqing Town) to 0.00333 (Sihai Town), with a relatively small coefficient of variation (ratio of standard deviation to mean) of 0.212. The above analyses indicate that the CHC and CHS accessibility is relatively even at the town-level, but shows significant spatial variations in certain towns.

**Fig. 8:**
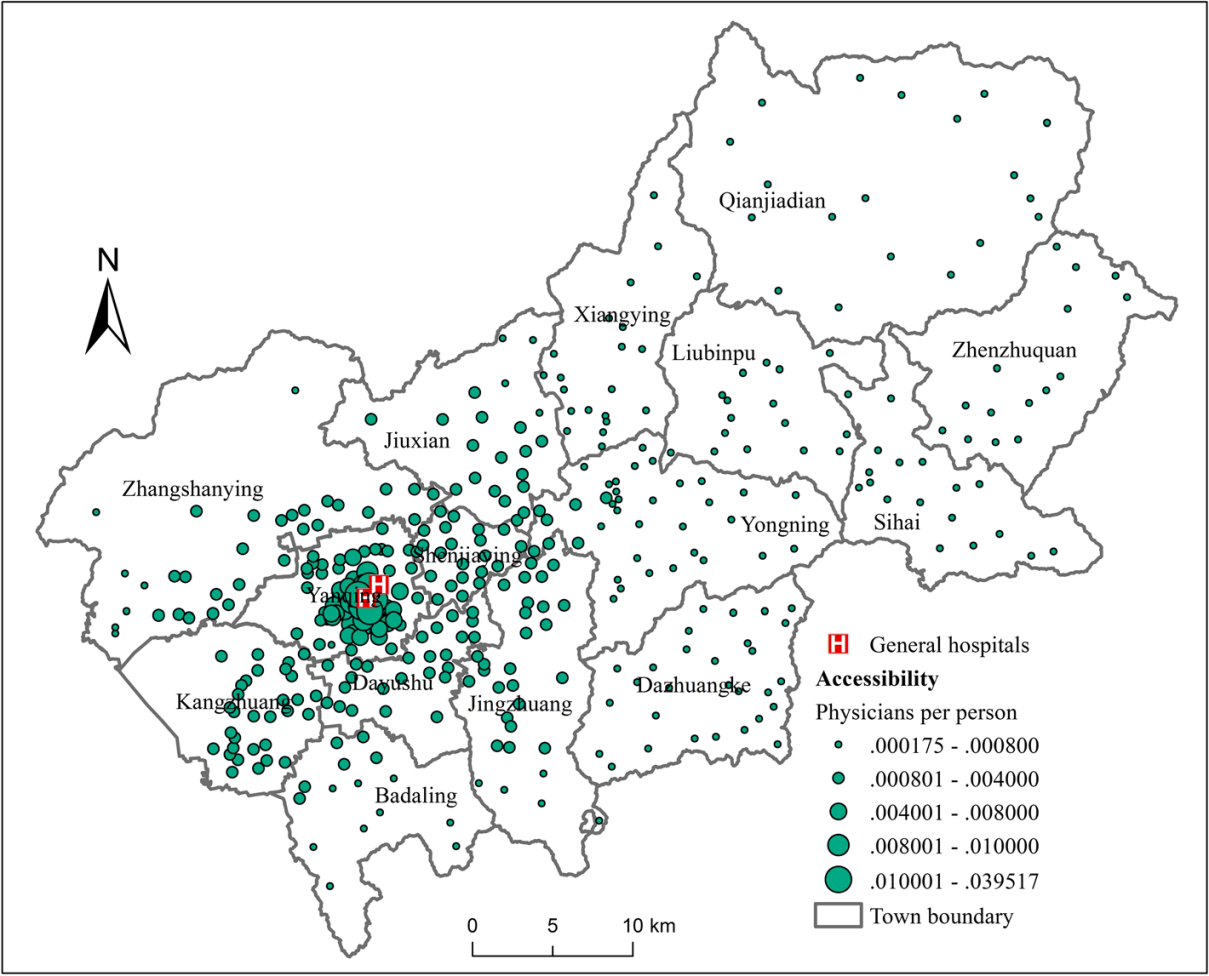
Accessibility to GHs in Yanqing District when *β* = 1

Differing from the distribution of CHC and CHS accessibility, the distribution of accessibility to GHs shows a significant uneven pattern, decreasing gradually outwards from the district center (Fig. 8). The reason is that both GHs are located in the district center, which is in the Yanqing Town. Accordingly, as shown in Fig. 7, the GH accessibilities of the towns surrounding the district center are higher than those in the eastern part of the district, which are relatively far away from the district center. The weighted average GH accessibility of each town ranges from 0.00022 (Qianjiadian Town) to 0.00716 (Yanqing Town). The coefficient of variation of town-level GH accessibility is 1.299, indicating a relatively high variation of GH accessibility among towns.

The overall accessibility including CHC, CHS and GH accessibility is also calculated for each town (Fig. 7). The overall accessibility of Yanqing Town is much larger than other towns, while the overall accessibility of the other 14 towns is relatively even. The coefficient of variation of town-level overall accessibility is 0.378. If Yanqing Town is excluded, the coefficient of variation of overall accessibility sharply decreases to 0.169, which is even smaller than the coefficient of variation of CHC and CHS accessibility. In a word, the small quantity and highly uneven distribution of GHs result in an obvious disparity in GH accessibility, which is the main cause of the disparity in overall accessibility.

### Comparison of accessibility with or without constraint of administrative boundary

**Fig. 9:**
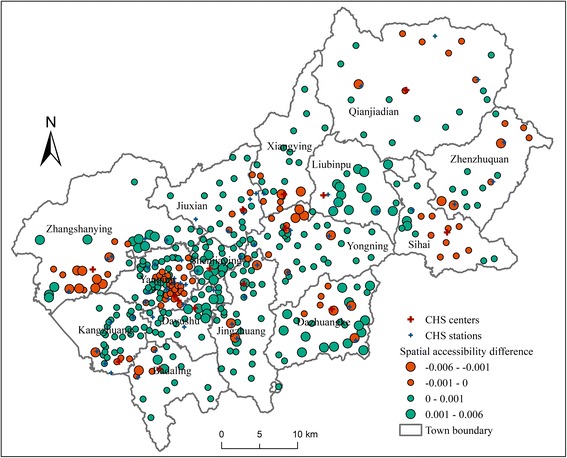
Difference between spatial accessibility with or without constraint of administrative boundary

Figure 9 shows the difference between spatial accessibility with or without constraint of administrative boundary (the with-constraint accessibility minus the without-constraint accessibility). The difference ranges from −0.006 to 0.006. Considering the mean of the without-constraint accessibility to CHCs and CHSs is 0.00149 and the range is from 0 to 0.02, this difference between two scenarios is relatively large. That is to say, the constraint of administrative boundary has a significant impact on healthcare accessibility.

Since the townships have quite different areas and their shapes are highly irregular, the distribution of accessibility difference does not show a regular pattern. In some townships, the accessibility differences of the villages that are close to the boundary are negative. In some other townships, the negative accessibility differences appear in the central villages. Therefore, if the constraint of administrative boundary is neglected in accessibility analysis in a case where this constraint actually exists, the bias of the accessibility result may exist. It is difficult to judge where the bias is positive and where the bias is negative. As our results show, however, the degree of this bias can be non-negligible. Therefore, the improvement made by this study in modeling constraint of administrative boundary in healthcare accessibility analysis is proven to be significant.

## Discussions

The main goal of this study is to develop a modified form of 2SFCA taking into account the constraint of administrative boundary on healthcare accessibility. The modification made by the proposed model is with respect to the definition on catchment areas of healthcare facilities. In the existing definitions of catchment areas in spatial accessibility literature, interactions between demanders and facilities can cross administrative boundaries [9, 11, 23]. The only exception is the measurement of supply-demand ratio, which calculates ratio of supply to demand in a certain region, usually administrative units [1]. However, the supply-demand ratio cannot reflect the impact of spatial separation between demanders and facilities, and is unable to measure the disparity in accessibility within each administrative unit. Moreover, in the supply-demand ratio method, only the administrative boundaries of demand nodes are considered having constraint effects on accessibility. In practice, as demonstrated in the second section as well as this case study, there may be multi-level administrative boundaries that affect the utilization behavior of healthcare services. The constraint of administrative boundary may also exist in other public services, e.g. education facilities. Therefore, the modified 2SFCA model proposed in this study can contribute for measuring the spatial accessibility of various types of public services.

In addition, as this case study in Yanqing District, Beijing, China shows, the modified 2SFCA method produces significantly different results with the traditional 2SFCA method as expected. The difference between the accessibility scores of the modified and traditional 2SFCA method shows an irregular pattern, due to the irregular shapes of administrative boundaries. The difference verifies the validity of the modified model in empirical studies. In cases where the constraint of administrative boundary on public services utilization behavior exists, traditional 2SFCA and its existing variants would lead to biased results. However, it should be noted that the modified 2SFCA method is a complement rather than a substitute to the existing methods. The modified model is only superior in situations with constraint of administrative boundary.

Existing studies have applied the potential model [28], 2SFCA [29], Enhanced 2SFCA [30] or Kernel Density 2SFCA [31] to measure spatial accessibility to healthcare services in China. None of these studies have considered the constraint effect of administrative boundary on healthcare accessibility. Our findings suggest that the results of these studies may be biased, due to the lack of the constraint of administrative boundary. The modified 2SFCA method proposed in this study can improve the measurement of healthcare accessibility in China.

Based on the empirical results, discussions on the spatial configuration of healthcare facilities can also be conducted. The GHs are aimed to provide healthcare services for the whole district, while the CHCs and CHSs provide services for the villages in each town. The CHCs are usually located close to the town governments. The CHSs are configured in certain villages. The sizes of CHCs are usually much larger than the CHSs. The sizes of CHSs are relatively even with several physicians at each station.

As for the CHSs, more attention should be paid to the towns with significant spatial difference of CHC and CHS accessibility, such as Qianjiadian, Zhenzhuquan, Xiangying, Jiuxian, Yongning and Jingzhuang. The spatial differences in CHC and CHS accessibility in these towns are mainly related to the distribution of CHSs, since the locations of CHCs are relatively fixed near the town governments. To reduce the spatial differences in CHC and CHS accessibility in these towns, more CHSs should be increased and dispersedly distributed in villages far away from existing CHSs as well as CHCs.

Since the capacity of CHSs is negligible compared with CHCs, the town-level CHC and CHS accessibility is mainly related to the sizes of the CHCs. In addition, the GHs are the dominate healthcare facilities in Yanqing District, but the distribution of accessibility to GHs is quite uneven due to the concentrated distribution of GHs. Therefore, the variation in overall healthcare accessibility at the town-level could be reduced in two ways. First, the sizes of CHCs in towns with small accessibility scores should be expanded. Second, new GHs can be built in the eastern part of Yanqing District. Taking into account the economic development levels and transportation conditions in the eastern towns, Yongning Town could be a potential location for a newly-built GH, which can significantly improve the healthcare accessibility in the eastern part of Yanqing District.

## Conclusions

This study has proposed a modification of the 2SFCA method for measuring spatial accessibility to healthcare services with constraint of administrative boundary. This modification could be helpful in cases where the administrative boundaries have constraint effect on the catchment areas of healthcare facilities and other types of public facilities.

The method has been applied in a case study of the healthcare services in Yanqing District of Beijing, China. The results show that the constraint of administrative boundary has a significant impact on healthcare accessibility, and confirm the significance of the modification proposed by this study. The results can also contribute for the planning-making of healthcare service resources allocation in the study area. Discussions have been conducted on how to optimize the spatial configuration of hierarchical healthcare facilities in order to improve the spatial equity of healthcare accessibility in the study area. At the town-level, the improvement of equity in healthcare accessibility could be achieved in two ways. First, the sizes of CHCs in towns with small accessibility scores should be expanded. Second, new GHs can be built in the eastern part of Yanqing District (e.g. in Yongning Town). Within each town, to improve the equity in healthcare accessibility, CHSs should be expanded or newly built in the periphery villages.

There are still some limitations in this study. Only road network distance is used to measure travel cost due to the lack of data with respect to actual travel time. Actual travel time OD matrix considering various transportation modes can make the results more accurate. The potential diversity of healthcare service demanders (e.g. different age groups) is also not involved in this study.

## Abbreviations

2SFCA: Two-step floating catchment area
CHC: Community healthcare center
CHS: Community healthcare station
G2SFCA: generalized 2SFCA
Gc: Gini coefficient
GH: General hospital

## References

[1] Wang F (2015). Quantitative methods and socio-economic applications in GIS.

[2] McGrail MR (2015). Spatial accessibility of primary health care utilising the two step floating catchment area method: an assessment of recent improvements. Int J Health Geogr.

[3] Guagliardo MF (2004). Spatial accessibility of primary care: concepts, methods and challenges. Int J Health Geogr.

[4] Wang F (2012). Measurement, optimization and impact of healthcare accessibility: a methodological review. Ann Assoc Am Geogr.

[5] Yang D, Goerge R, Mullner R (2006). Comparing GIS-based methods of measuring spatial accessibility to health services. J Med Syst.

[6] Cheng Y, Wang J, Rosenberg MW (2012). Spatial access to residential care resources in Beijing, China. Int J Health Geogr.

[7] Onega T, Duell EJ, Shi X (2008). Geographic access to cancer care in the U.S. Cancer.

[8] Hansen WG (1959). How accessibility shapes land use. J Am Inst Plann.

[9] Joseph AE, Bantock PR (1984). Measuring potential physical accessibility to general practitioners in rural areas: a method and case study. Soc Sci Med.

[10] Radke J, Spatial Decompositions ML (2000). Modeling and mapping service regions to predict access to social programs. Geographic. Inf Sci.

[11] Luo W, Wang F (2003). Measures of spatial accessibility to health care in a GIS environment: synthesis and a case study in the Chicago region. Environment and Planning B: Planning and Design..

[12] Luo W, Qi Y (2009). An enhanced two-step floating catchment area (E2SFCA) method for measuring spatial accessibility to primary care physicians. Health & Place..

[13] Dai D (2011). Racial/ethnic and socioeconomic disparities in urban green space accessibility: where to intervene?. Landsc Urban Plan.

[14] Dai D, Wang F (2011). Geographic disparities in accessibility to food stores in southwest Mississippi. Environment and Planning B: Planning and Design.

[15] Luo W, Whippo T (2012). Variable catchment sizes for the two-step floating catchment area (2SFCA) method. Health & Plac.

[16] McGrail MR, Humphreys JS (2014). Measuring spatial accessibility to primary health care services: Utilising dynamic catchment sizes. Appl Geogr.

[17] Tao Z, Cheng Y, Dai T (2014). Measuring spatial accessibility to residential care facilities in Beijing. Prog Geogr.

[18] Wan N, Zou B, Sternberg TA (2012). Three-step floating catchment area method for analyzing spatial access to health services. Int J Geogr Inf Sci.

[19] Delamater PL (2013). Spatial accessibility in suboptimally configured health care systems: a modified two-step floating catchment area (M2SFCA) metric. Health & Place..

[20] Luo J (2014). Integrating the huff model and floating catchment area methods to analyze spatial access to healthcare services. Trans GIS.

[21] Mao L, Nekorchuk D (2013). Measuring spatial accessibility to healthcare for populations with multiple transportation modes. Health & Place.

[22] Langford M, Higgs G, Fry R (2016). Multi-modal two-step floating catchment area analysis of primary health care accessibility. Health & Place..

[23] Jamtsho S, Corner R, Dewan A (2015). Spatio-temporal analysis of spatial accessibility to primary health care in Bhutan. International Journal of Geo-Information.

[24] Huotari T, Antikainen H, Keistinen T (2017). Accessibility of tertiary hospitals in Finland: a comparison of administrative and normative catchment areas. Soc Sci Med.

[25] Tao Z, Cheng Y, Dai T (2014). Spatial optimization of residential care facility locations in Beijing, China: maximum equity in accessibility. Int J Health Geogr.

[26] Wang F, Tang Q (2013). Planning toward equal accessibility to services: a quadratic programming approach. Environment and Planning B: Planning and Design..

[27] Health and Family Planning Commission of Beijing Yanqing District. 2016. http://www.yqwjw.gov.cn/yqwjw/fw/wyjy/yqdyy/yyzn/index.html. Accessed 5 May 2017. (In Chinese).

[28] Song Z, Chen W (2009). Measuring spatial accessibility to health care facilities based on potential model. Prog Geogr.

[29] Deng L, Shao J, Guo Y (2015). Spatial accessibility of medical services in mountainous regions based on modified two-step floating catchment area method: a case study of Shizhu County. Chongqing Progress in Geography.

[30] Hu R, Dong S, Zhao Y (2013). Assessing potential spatial accessibility of health services in rural China: a case study of Donghai county. Int J Equity Health.

[31] Cheng G, Zeng X, Duan L (2016). Spatial difference analysis for accessibility to high level hospitals based on travel time in Shenzhen, China. Habitat International.

